# Architecture and modular assembly of *Sulfolobus* S-layers revealed by electron cryotomography

**DOI:** 10.1073/pnas.1911262116

**Published:** 2019-11-25

**Authors:** Lavinia Gambelli, Benjamin H. Meyer, Mathew McLaren, Kelly Sanders, Tessa E. F. Quax, Vicki A. M. Gold, Sonja-Verena Albers, Bertram Daum

**Affiliations:** ^a^Living Systems Institute, University of Exeter, Exeter EX4 4QD, United Kingdom;; ^b^College of Engineering, Mathematics and Physical Sciences, University of Exeter, Exeter EX4 4QF, United Kingdom;; ^c^Molecular Enzymology, Faculty for Chemistry, University of Duisburg-Essen, 45141 Essen, Germany;; ^d^College of Life and Environmental Sciences, University of Exeter, Exeter EX4 4QD, United Kingdom;; ^e^Institute of Biology II, Molecular Biology of Archaea, University of Freiburg, 79104 Freiburg, Germany

**Keywords:** S-layers, electron cryotomography, *Sulfolobus*, archaea, subtomogram averaging

## Abstract

Surface protein layers (S-layers) often form the only structural component of the archaeal cell wall and are therefore important for cell survival. S-layers have a plethora of cellular functions including maintenance of cell shape, osmotic, and mechanical stability, the formation of a semipermeable protective barrier around the cell, and cell–cell interaction, as well as surface adhesion. Despite the central importance of S-layers for archaeal life, their 3-dimensional (3D) architecture is still poorly understood. Here we present detailed 3D electron cryomicroscopy maps of archaeal S-layers from 3 different *Sulfolobus* strains. We were able to pinpoint the positions and determine the structure of the 2 subunits SlaA and SlaB. We also present a model describing the assembly of the mature S-layer.

## Significance

Many bacteria and most archaea are enveloped in S-layers, protective lattices of proteins that are among the most abundant on earth. S-layers define both the cell’s shape and periplasmic space, and play essential roles in cell division, adhesion, biofilm formation, protection against harsh environments and phages, and comprise important virulence factors in pathogenic bacteria. Despite their importance, structural information about archaeal S-layers is sparse. Here, we describe in situ structural data on archaeal S-layers by cutting-edge electron cryotomography. Our results shed light on the function and evolution of archaeal cell walls and thus our understanding of microbial life. They will also inform approaches in nanobiotechnology aiming to engineer S-layers for a vast array of applications.

Surface protein layers (S-layers) encage many bacteria and archaea and are composed of surface proteins that are often glycosylated. These proteins arrange into flexible, porous, yet highly stable lattices that form cage-like coats around the plasma membrane. In bacteria, S-layers are anchored to the peptidoglycan or the outer membrane. In archaea, S-layers can be either incorporated into the periplasmatic polysaccharide layers, such as pseudomurein and methanochondroitin, or simply integrated into the cytoplasmic membrane. In most cases, S-layers form ordered 2-dimensional arrays and serve a variety of functions, which are thought to be specific to genera or groups of organisms sharing the same environment (1–3).

For archaea, S-layers are of particular importance as they often comprise the only cell wall component. They therefore define cellular shape and provide osmotic, thermal and mechanical stability (2). In addition, in vitro experiments have shown that S-layers change the physical and biochemical properties of lipid layers, rendering them less flexible, less fluid, more stable and heat-resistant, and possibly more resistant to hydrostatic pressure. Furthermore, it has been suggested that S-layers provide protection against immunological defense systems and viruses, act as pathogenic virulence factors, serve as phage receptors, promote surface adhesion, establish a quasi-periplasmic space, provide anchoring scaffolds for membrane proteins, sequester ions, and facilitate biomineralization (2). S-layers are intrinsically capable of self-assembly in vitro, resulting in tube-like, spherical 2D crystals (4). S-layers are therefore highly interesting for various applications in nanotechnology (5).

*Sulfolobus* is a genus of hyperthermophilic acidophilic archaea, which grow in low-pH terrestrial hot springs at 75–80 °C all around the globe. They are well-established model organisms, as they can be relatively easily cultured in the laboratory, and the genomes of a number of strains have been sequenced (6–8). The S-layer of all *Sulfolobus* species consists of 2 proteins, SlaA and SlaB. Biochemical analysis of SlaA and SlaB from different *Sulfolobus* species revealed molecular masses ranging from 120 kDa to 180 kDa or from 40 kDa to 45 kDa, respectively (9, 10). Comparative sequence analysis and molecular modeling of SlaB revealed that it exists in 2 species-dependent variants. In *S. ambivalens*, *S. acidocaldarius*, *S. tokodaii*, and *S. sedula*, SlaB is comprised of an N-terminal Sec-dependent signal sequence, followed by 3 consecutive β-sandwich domains, an α-helical coiled-coil domain, and 1 C-terminal transmembrane helix. In contrast, the sequences of *S. solfataricus* and *S. islandicus* are shorter by 1 β-sandwich domain (10). Interestingly, it was recently shown by negative stain electron microscopy (EM) that SlaB knockout strains of *S. islandicus* still assemble partial S-layers, consisting of only SlaA (11, 12).

In contrast, SlaA was predicted to be a soluble protein rich in β-strands and to form the outer region of the *Sulfolobus* S-layer (10). Deletion mutants of SlaA led to deformed cells without a distinctive cell envelope (13).

So far, the structure of the *Sulfolobus* S-layer has only been inferred by early 2D crystallography of negatively stained and isolated S-layers. These data suggested that *Sulfolobus* S-layers adopt a structurally conserved lattice with p3 symmetry, which encompasses 4.5 nm triangular and 8 nm hexagonal pores at a 21 nm distance (14, 15). However, as detailed 3-dimensional (3D) maps were so far unavailable, it has been unclear how SlaA und SlaB assemble into the final S-layer structure.

Using electron cryotomography (cryoET) and subtomogram averaging (STA), we obtained 3D cryoET maps of S-layers from 3 *Sulfolobus* species at unprecedented resolution. Through difference maps of fully assembled and SlaB-depleted S-layers, we were able to unambiguously pinpoint the positions of the component subunits SlaA and SlaB. Based on these experiments, we present a 3D model describing their assembly. In addition, our data reveal that strain-specific variants of SlaB lead to marked differences in the outward facing S-layer surface. We also find that SlaB is not required for SlaA S-layer assembly in vitro, which has important implications about the function of both S-layer components.

## Results

### In Situ Structure of the *Sulfolobus* S-Layer.

To obtain a detailed understanding of the molecular architecture of the archaeal S-layer, we chose the *Sulfolobus* species *S. islandicus (Sisl)*, *S. solfataricus (Ssol)*, and *S. acidocaldarius (Saci)*. In *Sisl* and *Ssol* the S-layer subunit proteins SlaA and SlaB share 87.4 and 87.7% sequence identity (*SI Appendix*, Fig. S1) and are thus virtually identical. In contrast, SlaA and SlaB from *Saci* show only ∼24 or ∼25% identity with *Sisl/Ssol* (*SI Appendix*, Fig. S2) and are thus far less conserved between those species. Moreover, SlaB from *Saci and Sisl/Ssol* have previously been proposed to represent 2 different structural “families” with respect to SlaB, which exists as a long form in *Saci* and a short form in *Ssol* and *Sisl* (10). We hypothesized that the 2 different variants of SlaA and SlaB may cause distinct S-layer geometries in *Saci* when compared to *Sisl* or *Ssol*.

**Fig. 1.**
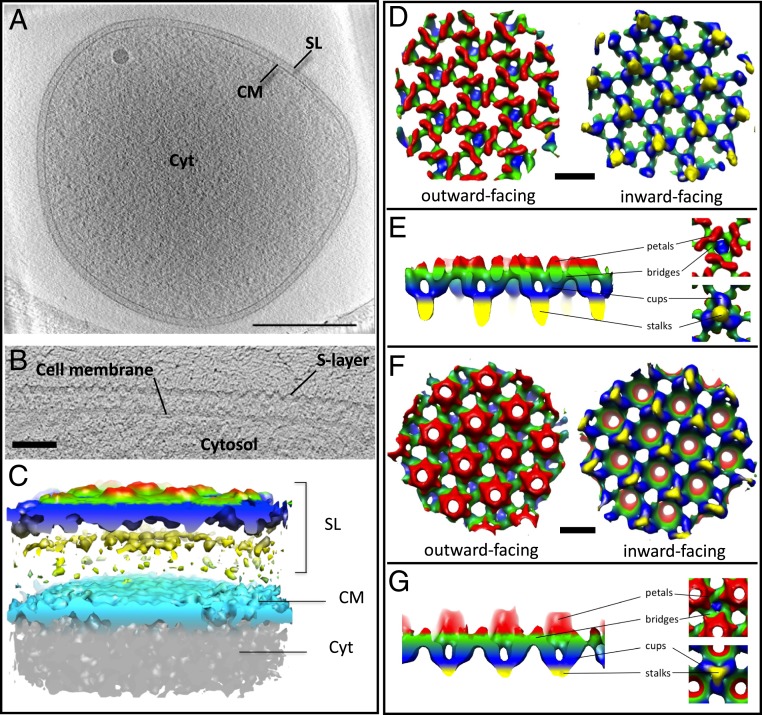
Cryoelectron tomography of *Sulfolobus* cells. (*A*) Tomographic slice through a *Sisl* cell. (Scale bar, *A*, 500 nm.) (*B*) Cross-section through the cell membrane and S-layer. (Scale bar, 50 nm.) (*C*) Segmented surface representation of the *Sisl* S-layer. (*D*–*G*) Subtomogram averages of *Saci* (*D* and *E*) and *Sisl* (*F* and *G*) S-layers. Maps are colored by proximity to the membrane plane. Yellow, proximal; red, distal. (Scale bars, *D* and *F*, 20 nm.)

To prepare cells for cryoET, cellular suspensions were plunge-frozen on holy carbon grids and investigated in the electron microscope. The majority of cells were surrounded by intact S-layers and membranes and showed various degrees of cytoplasmic density, which may either be a result of different metabolic states of slight cytosolic leakage during the sample preparation procedure. Tomographic tilt series were collected of cells with low cytosolic density, which were more transparent to the electron beam and thus resulted in tomograms with better signal-to-noise ratio (Fig. 1*A*).

In tomographic reconstructions, cells appeared disk-shaped with a diameter of up to 2 μm and a thickness of 250–300 nm (Fig. 1*A*). Since *Sulfolobus* cells are usually roughly spherical, this suggests that the cells had been compressed due to surface tension of the buffer during the plunge-freezing procedure. In tomographic cross-sections, 2 layers confining the cells were distinguished (Fig. 1*A*). Whereas the inner layer, the membrane, was smooth, the outer S-layer had a corrugated appearance (Fig. 1*B*).

Tomographic sections parallel to the plane of the S-layers clearly showed that they indeed form regular 2D arrays, as confirmed by power spectra of the respective tomographic slices. These power spectra showed clear spots up to the third order and indicated a lattice with p3 symmetry (*SI Appendix*, Fig. S3). The fuzzy spots indicated that the S-layers were not perfectly crystalline. This was expected, as roughly round shapes cannot be contained in a hexagonal lattice unless defects are included (16). Moreover, gaps in the lattice are needed to accommodate surface filaments such as archaella (17) or to allow the cells to grow and divide. In tomographic slices perpendicular to the membrane plane, S-layers formed regular, corrugated canopy-like arrays at a center-to-center distance of ∼30 nm from the membrane (Fig. 1*B*).

To obtain structural information of these S-layers in situ, subvolumes were cut from tomograms of *Sisl* and *Saci*, in which the cell surface was clearly resolved. Subsequently, those subvolumes were aligned and averaged in PEET. This resulted in 3D maps at ∼40 Å and 56 Å resolution for *Sisl* and *Saci*, respectively (*SI Appendix*, Fig. S5). Both S-layer maps revealed a perforated 2D protein lattice with p3 symmetry (Fig. 1 and *SI Appendix*, Figs.S4 and S5). For *Sisl*, unit cell dimensions were ∼21.9 × 20.9 nm including an angle of 120°, and for *Saci* the unit cell measured ∼23.9 × 23.6 nm with an angle of 120°. The unit cell of the *Saci* S-layer is therefore roughly 10% larger than that of *Sisl.* The total height of the maps was ∼25 nm for *Sisl* and ∼28 nm for *Saci*, respectively (Fig. 1 *D*–*G*). The averaged S-layer units were mostly aligned perpendicular to the electron beam, and the maximum tilt angles used for the collection of tomographic tilt series were limited to ±62°. Due to the missing wedge, the resulting maps appear stretched in *z*, roughly by a factor of 2.

Our 3D maps revealed structurally conserved and unique features for both S-layers (Fig. 1 *D*–*G*). In particular, the organization of the inward facing domains of the S-layer of both species is generally the same. In either case, they consist of trimeric stalks (Fig. 1, blue and yellow). These stalks support an outer canopy-like assembly (Fig. 1 *D*–*G*, green and red). At their membrane-proximal parts, each of the trimeric stalks merge into a single protrusion, giving them a tulip-like shape (Fig. 1 *E* and *G*, blue and yellow). This protrusion most likely forms the membrane anchor of the S-layer. Distal to the membrane, the stalks are connected to the S-layer canopy via their trimeric domains.

The outer canopy forms the outward facing side of the S-layer and resides on top of the array of the trimeric stalks (Fig. 1 *D*–*G*). In *Sisl* and *Saci* the base of the canopy appears to be cross-linked by a protein network, which has roughly the same in-plane geometry in both species (Fig. 1 *D*–*G*, green). In cross-section perpendicular to plane of the S-layers, this network forms a continuous band throughout the entire structure and includes repeating triangular and hexagonal pores. Two types of triangular pores can be distinguished, of which one is situated on top of a trimeric stalk (Fig. 1 *E* and *G*, blue), while the other is not. The triangular pores with and without stalk alternate throughout the lattice (Fig. 1 *D*–*G*).

The outward facing part of the canopy is distinct in both species. In *Sisl*, it is composed of an array of hexameric cone-shaped protrusions (Fig. 1*G*, red). Each of the cones encloses a hexagonal pore within the membrane. At their base, each cone is surrounded by 6 neighboring cones. The spaces between 3 cones contain the triangular pores (Fig. 1*G*).

In *Saci*, the most distal part of the outer layer is composed of elongated, petal-like structures (Fig. 1*D*, red). As in *Saci* these structures do not protrude as far from the general plane of the canopy, the outer S-layer face appears rather smooth compared to that of *Sisl*. As seen in Fig. 1*D*, each of these structures show a 2-fold symmetry around their long axis, which suggests that they are dimers.

### Structural Dissection of *Sulfolobus* S-Layers.

**Fig. 2.**
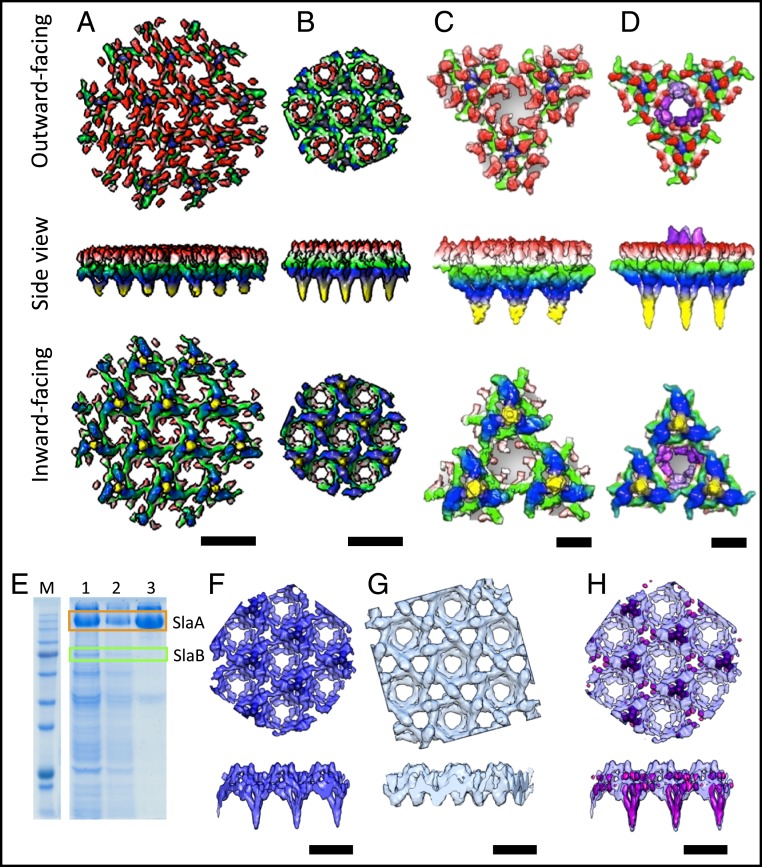
Structural dissection of isolated S-layers from *S. acidocaldarius* and *S. solfataricus*. (*A* and *B*) S-layer maps of *Saci* (*A*) and *Ssol* (*B*) at 23 Å or 16 Å resolution, respectively. (*C* and *D*) S-layer unit of *Saci* (*C*) and *Ssol* (*D*), including 3 stalks and a hexameric pore. Maps are colored by proximity to the membrane plane. Yellow, proximal; red, distal; purple, conical protrusions. (*E*) SDS/PAGE. M, marker; 1, washed once; 2, washed twice; 3, washed 3 times in detergent. (*F*) subtomogram average of fully assembled S-layer. (*G*) Subtomogram average of SlaB-depleted S-layer. (*H*) Difference map (pink) overlaid with the complete S-layer visualizes location of SlaB. (Scale bars, *A*, *B*, and *F*–*H*, 20 nm; *C* and *D*, 5 nm.)

To obtain higher-resolution maps for *Sulfolobus* S-layers and to investigate if the cell context is important to maintain correct S-layer assembly, we isolated S-layers—this time from *Saci* and *Ssol,* the latter of which was by sequence alignments predicted to have the same shortened domain structure as *Sisl.* Tomograms were recorded at optimized imaging conditions, and subtomogram averaging resulted in more detailed maps (Fig. 2) at 23 Å (*Saci*) and 16 Å (*Ssol*) resolution (*SI Appendix*, Fig. S5). The lattice parameters of the *Saci* S-layer remained unchanged, showing that their integrity does not crucially depend on the cellular context (Fig. 2*A*). Moreover, the map of the *Ssol* S-layer was virtually identical to that of *Sisl* in terms of lattice dimensions and outer canopy topology (Fig. 2*B*), indicating that 87% sequence identity conserves the general structural features.

To further investigate the structural differences between *Saci* and *Ssol*, we segmented each map into units composed of 3 stalks and the adjacent canopy (Fig. 2 *C* and *D*). As with *Sisl*, the S-layer of *Ssol* clearly showed a smaller unit size of 10% when compared to that of *Saci*. Furthermore, structural differences surrounding the hexagonal pores were resolved more clearly. Whereas in *Saci* each hexagonal pore is flanked by 6 inward curled protein domains, it is surrounded by 6 outward projecting densities in *Ssol,* giving rise to the cone-like assemblies.

### Pinpointing SlaA and SlaB.

In order to locate SlaA and SlaB within the *Sulfolobus* S-layer, we first removed the subunit SlaB from isolated S-layers using the detergent *N*-laurylsarcosine. After 3–4 repeated washing steps, this subunit ceased to be detectable by SDS/PAGE (Fig. 2*E*). CryoET and subtomogram averaging of the SlaB-depleted S-layer resulted in a map that lacked the trimeric stalks (Fig. 2*G*), leaving behind the porous canopy. This clearly indicates that SlaB constitutes the trimeric stalks that anchor the S-layer in the membrane. Consequently, the canopy must be formed by SlaA, which is in line with previous predictions (10).

To visualize the location and architecture of SlaB unambiguously, we calculated a difference map by subtracting the SlaB-depleted from the fully assembled map (Fig. 2*H*). This revealed that SlaB adopts a tripod-like shape, which is consistent with earlier sequence-based predictions that suggested that SlaB forms a trimer (10). In our structure, the 3 branches of each SlaB trimer are buried inside the SlaA canopy, while the “monomeric” stalk projects away from it, toward the membrane plane. In multitude, these pillars act to raise the SlaA canopy above the membrane. Interestingly, individual SlaB units are not in contact, but are linked to each other via the SlaA canopy network. Notably, the canopy structure of the SlaB-depleted S-layer was less well resolved than in the fully assembled control sample (Fig. 2*F*). This suggests that SlaB may act in reinforcing the stability of the SlaA network.

### Role of SlaB in S-Layer Assembly.

**Fig. 3.**
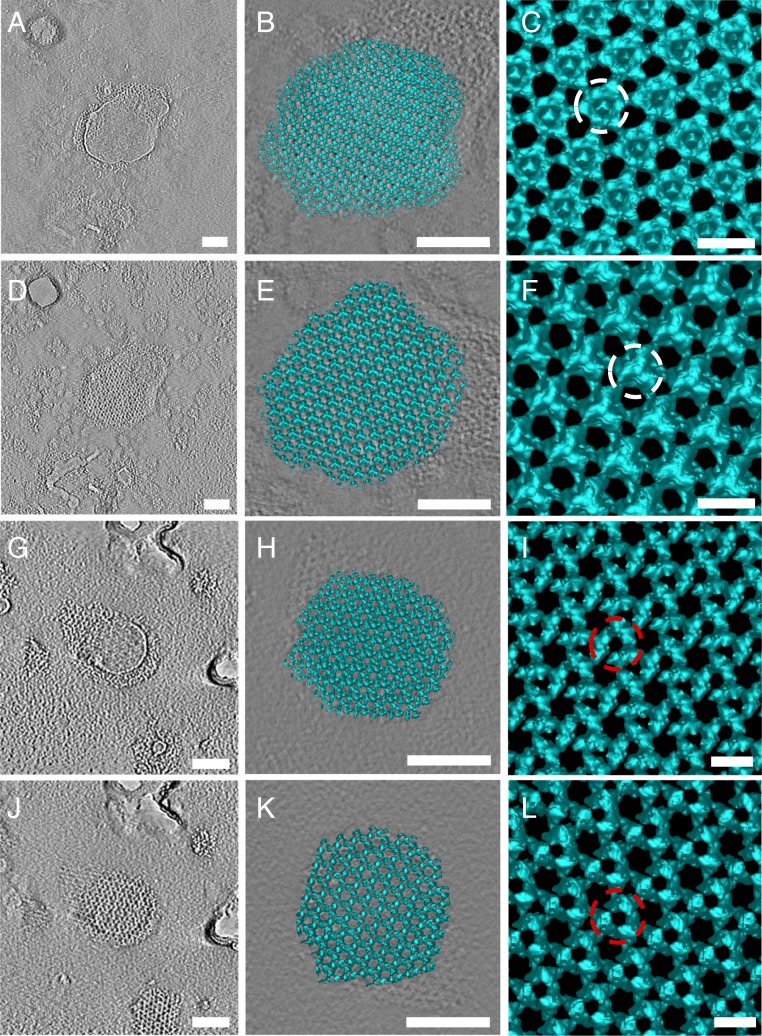
S-layers recrystallize with and without SlaB. (*A*–*F*) Reassembly of *Sulfolobus* S-layers from SlaA and SlaB. (*G*–*L*) Reassembly of S-layers from SlaA only. (*A*, *D*, *G*, and *J*) Tomographic slices. (*B*, *E*, *H*, and *K*) Three-dimensional surface representation. (*C*, *F*, *I*, and *L*) Subtomogram averages. White circle highlights presence of SlaB (*C* and *F*), and red circles highlight absence of SlaB (*I* and *L*). (Scale bars, *A*, *B*, *D*, *E*, *G*, *H*, *J*, and *K*, 100 nm; *C*, *F*, *I*, and *L*, 20 nm).

As the S-layer structure is maintained after removal of SlaB, we asked if SlaB proteins merely function as pillars for the SlaA lattice or if they also correct assembly of the outer canopy. To investigate this, we isolated S-layers from both *Saci* and *Ssol*, disassembled them by transfer into pH 10 buffer, and subsequently performed recrystallization by dialysis against water at pH 7 and incubation for 120 h. Inspection of the reaction containing both subunits in the electron microscope revealed patches of reassembled S-layers (Fig. 3 *A* and *D*). Subtomogram averaging showed that these S-layers had retained their original lattice dimensions (Fig. 3 *B* and *E*) and that the SlaA canopy as well as SlaB stalks could be identified (Fig. 3 *C* and *F*).

Interestingly, electron microscopy of the SlaB-depleted samples also revealed newly formed S-layers (Fig. 3 *G*, *H*, *J*, and *K*). Again, subtomogram averaging confirmed that the reassembled S-layers had adopted their original lattice parameters; however, this time without the SlaB stalks (Fig. 3 *I* and *L*). These observations indicate that SlaB is not involved in assembly process of the SlaA canopy and suggest that it mainly functions as a membrane anchor and distance ruler that determines the width of the periplasmic space.

### Assembly Model of the *Sulfolobus* S-Layer.

It has previously been shown biochemically and confirmed by us that SlaA forms a stable ∼250 kDa dimer in solution (10). In conjunction with early electron microscopy, it has also been predicted that this dimer forms the primary *Sulfolobus* S-layer building block (10, 15). This repeating dimer array is apparent in our subtomogram averages of negatively stained SlaA-only S-layers (Fig. 3*I*). To build a cryoEM model of the *Saci* and *Ssol* S-layers, we first segmented the SlaB-depleted subtomogram-averaging maps by the water-shedding method in Chimera (18). For guidance, we used the constraints of repeating dimeric SlaA densities that roughly fitted the molecular weight of 250 kDa, as well as our negative stain map in which the individual dimers were clearly visible (Fig. 3*I*). We then superimposed this segmentation with the fully assembled S-layer, which provided us with the positions of SlaB trimers.

**Fig. 4.**
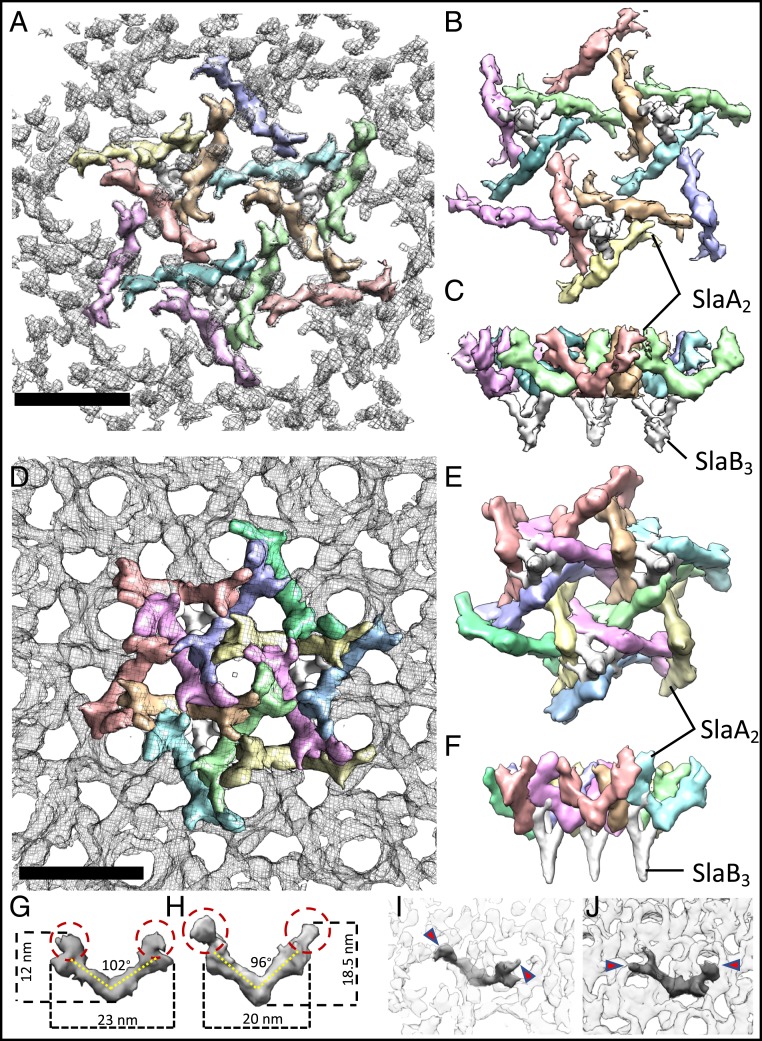
Assembly models for the *S. acidocaldarius* (*A*–*C*) and *S. solfataricus* (*D*–*F*) S-layers. (*A* and *D*) Outward facing surfaces (gray mesh) segmented into SlaA dimers (each dimer in a different color) and SlaB trimers (white). (*B* and *E*) Inward facing surface. (*C* and *F*) Side view, perpendicular to the membrane plane. (*G*–*J*) Comparison of the structures of SlaA from *Saci* (*G*) and *Ssol* (*H*) show differences in length, height, and angular shape of the dimer. (*I* and *J*) Location of 1 SlaA dimer within the S-layer of *Saci* (*I*) and *Ssol* (*J*). Red circles/arrowheads indicate apical domains that determine shape and topology of the hexameric pores. (Scale bars, 20 nm).

The resulting model shows the location of SlaA dimers and SlaB trimers within the assembled S-layers of *Saci* (Fig. 4 *A*–*C*) and *Ssol* (Fig. 4 *D*–*F*). In both cases, individual SlaA dimers adopt the shape of a boomerang. The elbow of each boomerang interacts with 1 of the 3 branches of a SlaB trimer, whereas both SlaA arms project outward. Thus, each SlaB trimer is bound to 3 SlaA dimers (SlaB_3_/3SlaA_2_), which intersect to form a triangular pore above each SlaB trimer. Three of these flower-shaped SlaB_3_/3SlaA_2_ surround 1 hexagonal pore, formed by 6 SlaA dimers. Herein, each of the 3 SlaB trimers contributes 2 SlaA dimers to the hexameric ring. At the same time, each SlaA dimer spans 2 neighboring hexameric pores. The second triangular pore type that does not coincide with a SlaB trimer is formed by 3 intersecting SlaA dimers at the interface of 3 hexagonal pores. In total, each SlaA dimer interacts with 4 SlaA dimers and 3 SlaB trimers. Therefore, SlaA dimers interlink into a tightly woven S-layer canopy that is stabilized by multiple interactions.

### SlaA Determines S-Layer Geometry and Topology.

To understand how *Saci* and *Ssol* assemble S-layers with similar geometry but different topology and unit cell size, we compared the structures of the SlaA proteins from each species. To this end, we extracted individual dimer densities from our segmented maps shown in Fig. 3.

This comparison revealed that while the overall shape of the dimers is the same, there are marked differences with respect to their horizontal and vertical dimensions, as well as the angles between both arms of each boomerang (Fig. 4 *G*–*J*). *Saci* SlaA measures 23 nm along its long axis, which corresponds with the size of the unit cell of the S-layer (Fig. 4*G*). This is to be expected, as this molecule spans to neighboring hexagonal S-layer pores (Fig. 4*I*). The corresponding portion of *Ssol* SlaA measures ∼20 nm (Fig. 4*H*), which is again in accordance with the 10% smaller assembled array (Fig. 4*J*). The length of the SlaA dimer therefore determines the size of the unit cell. The differences in length appear to be established by the angle between both arms of the molecule, which is 102° for the longer *Saci* SlaA and 96° for the shorter *Ssol* homolog. Interestingly, both SlaA variants also differ in an apical domain, which is curled inward in *Saci* and projects upward in *Ssol*. In assembly, these domains are responsible for the differences in the topology of both S-layers, as it is these domains that delineate the shallow hexagonal pores in S*aci* and the conical protrusions in *Ssol* (Fig. 4 *I* and *J*). Taken together, the strain-specific dimensions and surface morphology of *Sulfolobus* S-layers are determined by unique structural characteristics of SlaA, in particular its apical domains.

## Discussion

### Roles of SlaA and SlaB in S-Layer Assembly.

Previous studies employing biochemistry and negative stain electron microscopy showed that *Sulfolobus* S-layers consist of SlaA and SlaB proteins that assemble into a porous S-layer with P3 symmetry (10, 13). However, the position and organization of the individual subunits was so far unknown. Here we describe cryoET-based molecular maps of the *Sulfolobus* S-layer. We now unambiguously show that the membrane-proximal face of the S-layer consists of tripod-like SlaB trimers, which were previously (10, 14, 15) not clearly resolved. Our data also show that these SlaB trimers support the outer porous canopy of the S-layer, which is formed by an array of tightly interwoven boomerang-shaped SlaA dimers. Species-specific differences in S-layer topology are established by distinct structural features of SlaA.

**Fig. 5.**
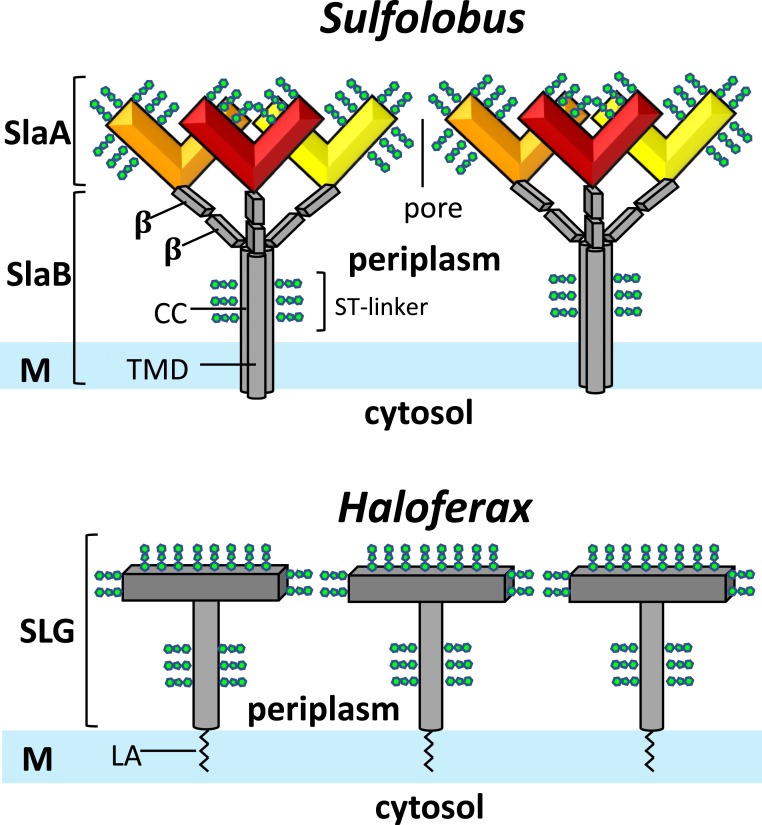
Model of the *Sulfolobus* S-layer compared to the single-subunit S-layer of haloarchaea. The *Sulfolobus* S-layer (*Upper*) consists of the 2 protein subunits: SlaA dimers (red, orange, yellow) form the outer S-layer canopy. Each SlaA protein is predicted to be rich in β-strands. The SlaA dimer has a boomerang-like shape, the angle of which determines the S-layer unit cell size. SlaB trimers (gray) form the membrane anchors of the S-layer. Each SlaB is predicted to consist of an N-terminal transmembrane domain (TMD), a coiled-coil domain (CC), and 2–3 C-terminal β-sandwich domains (β). SlaA and SlaB proteins are highly glycosylated (green). In contrast, the S-layers of many halophile archaea (such as that of *Haloferax volcanii*; *Bottom*) consist of multiple copies of only 1 S-layer glycoprotein (SLG) subunit and are inserted into the membrane by a posttranslationally added lipid anchor (LA).

While X-ray structures for neither of the component proteins are available, sequence predictions indicated that SlaA and SlaB contain Sec-dependent signal peptides and are thus translocated through the membrane by the general secretory pathway. In addition, glycoproteomic analysis showed that both proteins contain several sites for N- and O-linked glycosylation and are heavily glycosylated prior to assembly (19, 20) (Fig. 5). Moreover, SlaB has been predicted to consist of an N-terminal membrane-anchor, followed by coiled-coil domain including a serine and threonine-rich highly glycosylated region (ST linker) and 2–3 C-terminal β-sandwich domains (10) (Fig. 5). Upon trimerization of SlaB, 3 C-termini presumably form a trimeric coiled-coil, which most likely corresponds to the stalk-like base of each SlaB tripod (Fig. 5). The SlaB stalks in our maps appear shorter than expected for a coiled-coil of roughly 100 amino acids (10) in length. This is likely due to high flexibility in this region, which is thus mostly averaged out during our subtomogram-averaging procedure. The 3 protrusions of each SlaB tripod are likely formed by the predicted C-terminal β-sandwich domains (10) of the 3 SlaB proteins in the trimer, which therefore also form the interface with the overarching SlaA lattice (Fig. 5). However, due to the limited resolution of our maps, we were unable to distinguish between the length differences in the *Saci* and *Ssol* SlaB β-sandwich domains.

Our reassembly experiments reveal that SlaB is not required for SlaA self-assembly in vitro. This is consistent with recent whole-cell negative stain EM data, which showed that *Sisl* SlaB knockout species still assemble partial S-layer–like coats that consisted of only SlaA and were partially or completely detached from the cell membrane (11, 12). We conclude that the SlaA protein is the driving factor of S-layer assembly. In addition, our data show that strain-specific S-layer surface features, including lattice dimensions and topology, are determined by the shape of SlaA.

In contrast, SlaB’s main function is to serve as a membrane anchor, distance ruler, and to create the periplasmic space. This space has been proposed to have important roles in archaeal cell biology, including the coordination of membrane-embedded molecular machines such as the archaellum (17, 21) and various other archaeal surface structures (22). Secondarily, we observe that SlaA-only S-layers are slightly less ordered than their fully assembled counterparts, indicating that SlaB also increases the stability of the S-layer, presumably by cross-linking 3SlaA_2_ subcomplexes with each other.

The functional significance of the different S-layer topologies is largely unknown and awaits further exploration. It may be speculated that distinct S-layer surface structures have evolved to modulate the cell-adhesive properties of different archaeal species. In addition, S-layers may act as receptors in cell-cell recognition or phage infection (23), and we could show previously that archaeal viruses evolved elaborate cell entry and egress strategies to overcome the S-layer barrier (24–26). It is therefore conceivable that distinct S-layer topologies provide unique recognition tags for species-specific interaction and communication and that the evolution of new structures is a manifestation of the cell’s strategy to avoid viral infection (27). SlaB, in contrast, appears structurally more conserved than SlaA. Indeed, it has been shown previously that SlaB proteins have lower sequence variability than SlaA and their molecular masses differ less across different *Sulfolobus* species (10). This is likely due to the fact that SlaB interacts less with the extracellular medium and is thus less prone to environmentally related evolutionary pressures.

Recently, an additional paralogous SlaB gene product (M164_1049, SSO1175, or WP_011278654.1 in *Sisl*, *Ssol,* or *Saci*, respectively) was suggested to form another membrane-anchored protein component of the *Sulfolobus* S-layer (13). Double M164_1049 and SlaB mutants showed that no S-layer is present in *Sisl*, as opposed to single SlaB mutants where a partial coat of SlaA was observed. In addition, a single M164_1049 deletion mutant showed virtually no difference compared to the wild type. It was concluded that M164_1049 may aid in anchoring SlaA to the cytoplasmic membrane (13). Based on our maps, we cannot confirm the presence of this protein. As it is membrane-anchored, it likely resides near the membrane plane, the region which is averaged out in our data due to the highly flexible nature of the SlaB coiled-coil domain.

In contrast to *Sulfolobus,* many archaeal species possess only 1 S-layer protein. For example, in many haloarchaea, a single S-layer glycoprotein (gene product *csg1*/S-layer glycoprotein in *Haloferax volcanii*) combines membrane-anchoring and canopy-forming functions into 1 protein. Moreover, this protein is not membrane-integral but instead inserted to the bilayer via a C-terminal lipid anchor (Fig. 5) (28). While employing only 1 S-layer protein might be energetically more favorable, it is likely that using 2 increases the adaptability of the S-layer surface (SlaA) without compromising the membrane anchor (SlaB).

### Function of S-Layer Pores.

Among the most striking features of the *Saci* and *Ssol* S-layers are the circular apertures of ∼4.5 or ∼8 nm in diameter that repeat throughout the array. Similar porous, skeleton-like architectures have been reported for other S-layers such as that of the bacterium *Caulobacter crescentus* (29), highlighting that hexagonal symmetry and S-layer porosity are highly conserved traits throughout bacterial and archaeal species and thus provide a significant evolutionary advantage. It is likely that—similar to chainmail—this skeleton-like structure enhances S-layer flexibility, enabling it to coat cell bodies of various shape and allowing the cell to morph and divide. It is also tantalizing to assume that the particular shape and size of the pores has evolved to accommodate particular cellular filaments, such as adhesive pili or archaella. In contrast to this notion, we find that most known archaeal surface filaments, such as adhesive (AAP) pili (30, 31) and archaella (17, 32, 33), are over 10 nm in diameter and thus too wide to be accommodated by S-layer pores. Thus, the S-layer will have to be partially disassembled or adopt a different local geometry wherever these filaments emerge from the cell body (17). Finally, it is safe to assume that due to their porosity, S-layers provide a semipermeable barrier, similar to the outer membrane of Gram-negative bacteria, albeit with a more liberal molecular weight cutoff. At first glance, this cutoff appears to be determined by the pore diameter (<8 nm), which would allow a large variety of solutes, macromolecules, and even large proteins to pass. However, this notion may be deceptive, as both SlaA and SlaB are highly glycosylated (19, 20). While (likely due to their high flexibility) these posttranslational modifications are averaged out in our cryoEM maps, they are thought to cover much of the S-layer surface and thus possibly also project into the pores (Fig. 5). It is conceivable that these glycans would significantly lower the permeability of the S-layer pores to macromolecules, similar to the hydrogel found in nuclear pore complexes (34).

## Conclusions

We present detailed 3D models of S-layers of 3 different *Sulfolobus* species and pinpoint the location and organization of their component subunits SlaA and SlaB. We find that the structure of the SlaA dimer determines the unit cell size and topology of the S-layer, while SlaB anchors the S-layer in the plasma membrane and defines a pseudoperiplasmic space. Different S-layer topologies between species are likely the result of various evolutionary adaptations, including cell–cell interactive specificity and adhesive properties. Despite being an ubiquitous hallmark in archaea, many functional aspects of S-layers still need to be clarified through further structural investigation.

## Materials and Methods

### Strains and Growth Conditions.

The strains *Ssol* and *Saci* MW001 were grown in basal Brock medium* at pH 3 (35). The medium was supplemented with 0.1% (wt/vol) NZ-amine and 0.2% (wt/vol) dextrin just before inoculation. For growth of *Saci* MW001, 10 μg/mL uracil was added to the medium. *Sulfolobus* cultures were grown at 75 °C, 150 rpm, until an OD600 of >0.6 was reached. Cells were harvested by centrifugation at 5000 × *g* (Sorvall ST 8R) for 30 min and stored at −20 °C for subsequent use.

* Brock media contain (amount per liter): 1.3 g (NH_4_)_2_SO_4_, 0.28 g KH_2_PO_4_, 0.25 g MgSO_4_ × 7H_2_O, 0.07 g CaCl_2_ × 2H_2_O, 0.02 g FeCl_2_ × 4H_2_O, 1.8 mg MnCl_2_ × 4H_2_O, 4.5 mg Na_2_B_4_O_7_ × 10H_2_O, 0.22 mg ZnSO_4_ × 7H_2_O, 0.05 mg CuCl_2_ × 2H_2_O, 0.03 mg NaMoO_4_ × 2H_2_O, 0.03 mg VOSO_4_ × 2H_2_O, and 0.01 mg CoSO_4_ × 7H_2_O.

### S-Layer Isolation.

Cell pellets of frozen cells from a 50-mL culture were incubated and inverted on a rotator at 40 rpm (Stuart SB3) for 45 min at 37 °C, in 40 mL of buffer A (10 mM NaCl, 1 mM phenylmethylsulfonyl fluoride, 0.5% sodium lauroylsarcosine), with the addition of 10 μg/mL DNase I just prior to use. The samples were pelleted by centrifugation at 18,000 × *g* (Sorvall Legend XTR) for 30 min and subsequently resuspended in 1.5 mL buffer A, before further incubation and inversion at 37 °C, for 30 min. After centrifugation at 14,000 rpm for 30 min (Sorvall ST 8R), the pellet was purified by resuspension and incubation in 1.5 mL buffer B (10 mM NaCl, 0.5 mM MgSO_4_, 0.5% SDS) and rotated for 20 min at 37 °C, 40 rpm. To retain both SlaA and SlaB, only 1 wash was performed. To remove SlaB, washing with buffer B was repeated a further 3 times. Purified S-layer proteins were washed once with distilled water and stored at 4 °C. The removal of SlaB was confirmed by disassembly of an aliquot of the sample (as described below) and SDS/PAGE analysis

### Disassembly and Reassembly of S-Layers.

S-layers were disassembled by increasing the pH with the addition of 20 mM NaCO_3_, pH 10, 10 mM CaCl_2_ and incubation at 60 °C, 600 rpm (Thermomixer F1.5, Eppendorf) for 1 h. Disassembly was verified by SDS/PAGE analysis.

Reassembly was achieved by buffer exchange using 0.5-mL concentrators according to the manufacturer’s instructions (Pierce), with dH_2_O to reduce the pH to 7, followed by the addition of 10 mM CaCl_2_ and incubation at 60 °C for 120 h.

### Negative Stain Tomography.

A 3-μL sample of S-layers with and without SlaB was placed on glow-discharged carbon-coated copper grids (Agar Scientific) and incubated at room temperature for 1 min. The excess sample was removed by blotting, using filter paper (Whatman, GH healthcare). The specimens were stained using ammonium molybdate for 1 min. Grids were blotted dry with filter paper and let air dry completely. Single-axis tilt series (−60 to +60) were recorded on an FEI T12 electron microscope operated at 120 kV, using DigitalMicrograph (Gatan, Inc.).

Tilt series were reconstructed using the IMOD package (36), and tomograms were generated using the weighted back-projection algorithm.

### Electron Cryotomography.

Three microliters of S-layer suspension were applied to glow-discharged 300-mesh copper Quantifoil grids (R2/2, Quantifoil), blotted for 3–5 s, and rapidly injected into liquid ethane using a homemade plunge-freezer. Tomograms were recorded using a Polara G2 Tecnai (Thermo-Fisher) or a Jeol 3200 FSC transmission electron microscope (TEM) (JEOL), both based at the Max Planck Institute of Biophysics in Frankfurt, Germany. The microscopes were operated at 300 kV. Data were collected using a 4 × 4 k CCD camera or a 4 × 4 k K2 Summit direct electron detector (Gatan, Inc.) running in counting mode. Inelastically scattered electrons were removed using a Gatan Tridiem energy filter (Gatan, Inc.) for the Polara and an in-column energy filter for the JEOL microscope. Tilt series were collected in zero-loss mode using Digital Micrograph (Gatan) from −62° to +62° and in steps of 2°. For tomograms of whole cells, the Polara was used. The magnification was set to 34.000 (Sisl) × or 27.500 (Saci) × (resulting in a final pixel size of 0.709 nm (Sisl) or 1.073 nm (Saci) on the final image), and defocus values of 6–8 μm were applied. For isolated S-layers, the magnification used was 72,000 (final image pixel of 2.8 Å) on the Polara and 10,000 × (final pixel size 3.35 Å) on the JEOL. Defocus values of 2–4 μm were used. Whole-cell tomograms were recorded using a CCD camera with a total dose of 100 e^−^ Å^−2^. Tomograms of isolated S-layers were recorded using the K2 and in dose-fractionation mode at a dose rate of 8–10 e^−^ px^−1^ s^−1^ and a maximum total dose of 60 e^−^ Å^−2^. Dose-fractionated tilt images were aligned using an in-house script based on IMOD (36) programs, Contrast Transfer Function (CTF)-corrected and reconstructed into tomograms using the IMOD software package (36).

### Subtomogram Averaging.

To perform subtomogram averaging on the S-layer, a random grid of points was applied over the S-layer within a tomograms of *Sulfolobus* cells or isolated S-layers, which were previously binned 2-fold. Whole-cell tomograms were filtered by nonlinear anisotropic diffusion (NAD) (37) to enhance the contrast. Using PEET (38), subvolumes were extracted, aligned, and averaged. For the in situ S-layer average, 1,809 subvolumes (*Sisl*) and 2,068 subvolumes (*Saci*) of 60 × 60 × 60 pixels in dimension were used. For isolated S-layers, 325 subvolumes (*Saci*) and 5,561 subvolumes (*Ssol*) of 100 × 100 × 80 were used. p3 symmetry was applied to the final averages. S-layers were visualized and segmented using UCSF Chimera (18). The resolution of all averages was estimated based on the reflections in their respective power spectra calculated by IMOD (36). For the in situ structures this suggested a resolution of 56 Å for *Saci* and 40 Å for *Sisl*. For the maps of the isolated S-layers, 16 Å were measured for *Ssol* and 23 Å for *Saci* (*SI Appendix*, Fig. S5).

### Difference Maps and Assembly Models.

The difference maps were calculated and assembly models built in UCSF Chimera (18). To calculate the difference map between the fully assembled and SlaB-depleted S-layers, the *vop subtract* command was used. This resulted in clear densities for SlaB trimers. To build the assembly model of the SlaA canopy regions, semiautomated water-shedding segmentation was initially used on the subtomogram average of the SlaB-depleted S-layers. This approach segmented the map in equal-sized parts, which were then grouped to generate a p3 lattice of dimers. Individual SlaB and SlaA subunits were extracted as separate densities from the map and fitted into the fully assembled *Ssol and Saci* S-layers to yield the complete SlaA/SlaB assembly model.

### Sequence Alignments.

Protein sequences were sourced online using Uniprot (https://www.uniprot.org/) and multiple sequence alignments performed with the Praline Server (http://www.ibi.vu.nl/programs/pralinewww/).

### Data Availability.

The cryo-EM maps were deposited in the EM Data Resource (https://www.emdataresource.org/) with accession codes EMD-10459 (*Sisl* in situ), EMD-10460 (*Saci* in situ), EMD-10444 (*Saci* isolated), and EMD-10445 (*Ssol* isolated).

## Supplementary Material

Supplementary File
