# Supplementary material for: Architecture and modular assembly of Sulfolobus S-layers revealed by electron cryotomography
Source: Proc Natl Acad Sci U S A. 2019 Nov 25;116(50):25278–86. doi: 10.1073/pnas.1911262116 (PMC6911244; doi:10.1073/pnas.1911262116)
Supplement: Supplementary File [file pnas.1911262116.sapp.pdf]

Supplementary Appendix

Fig S1

A

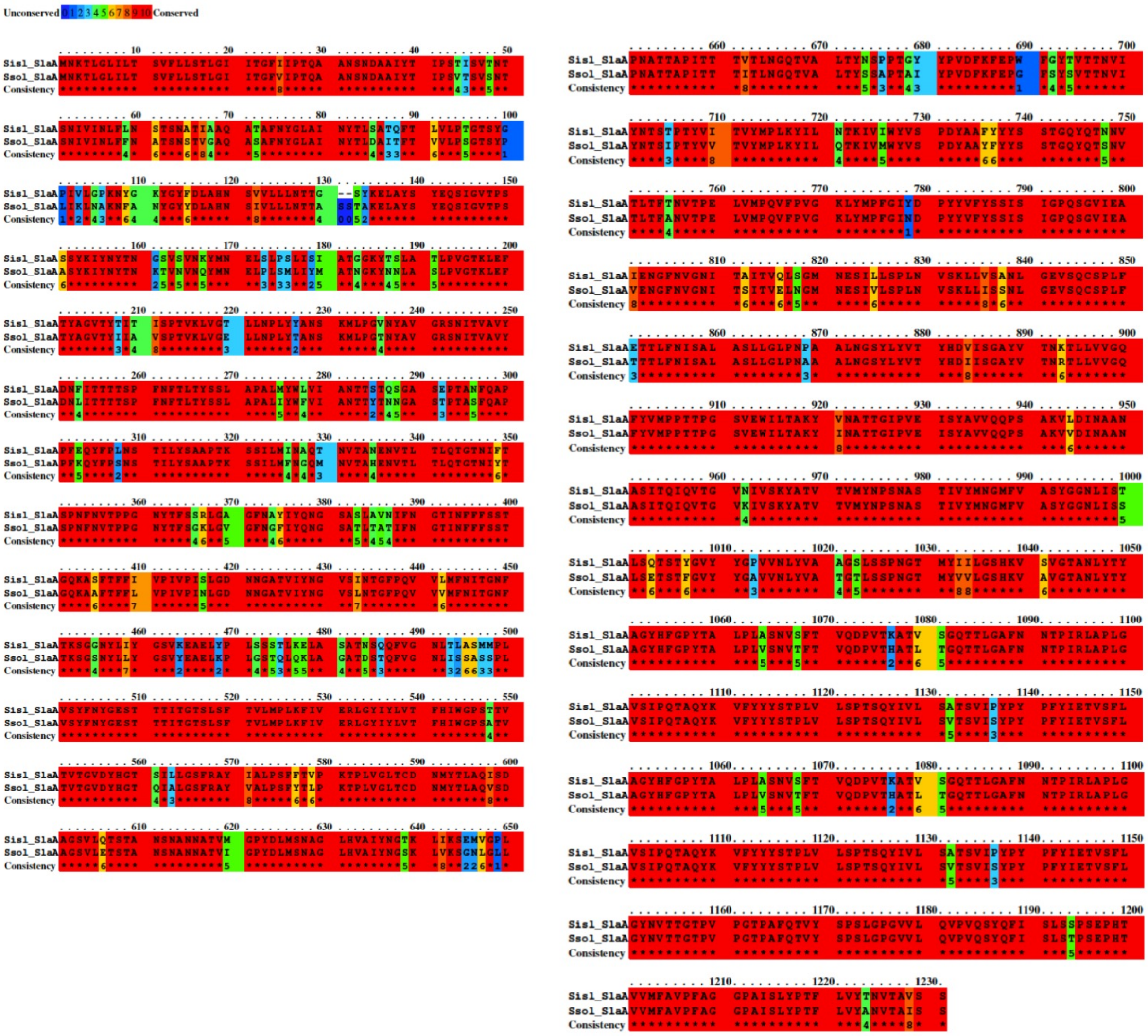

Fig S1

B

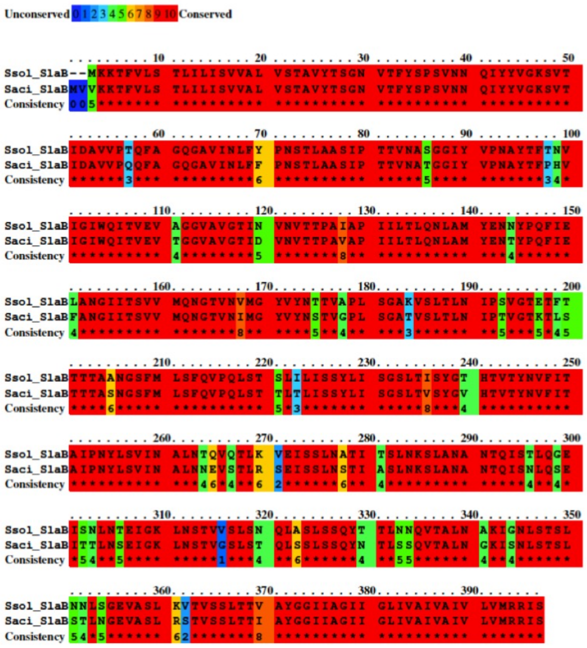

Figure S1 – Sequence alignment comparing SlaA (A) and SlaB (B) from *Sisl* and *Ssol*. The sequence identity for SlaA and SlaB is 87.4% and 87.7%, respectively.

Fig S2

A

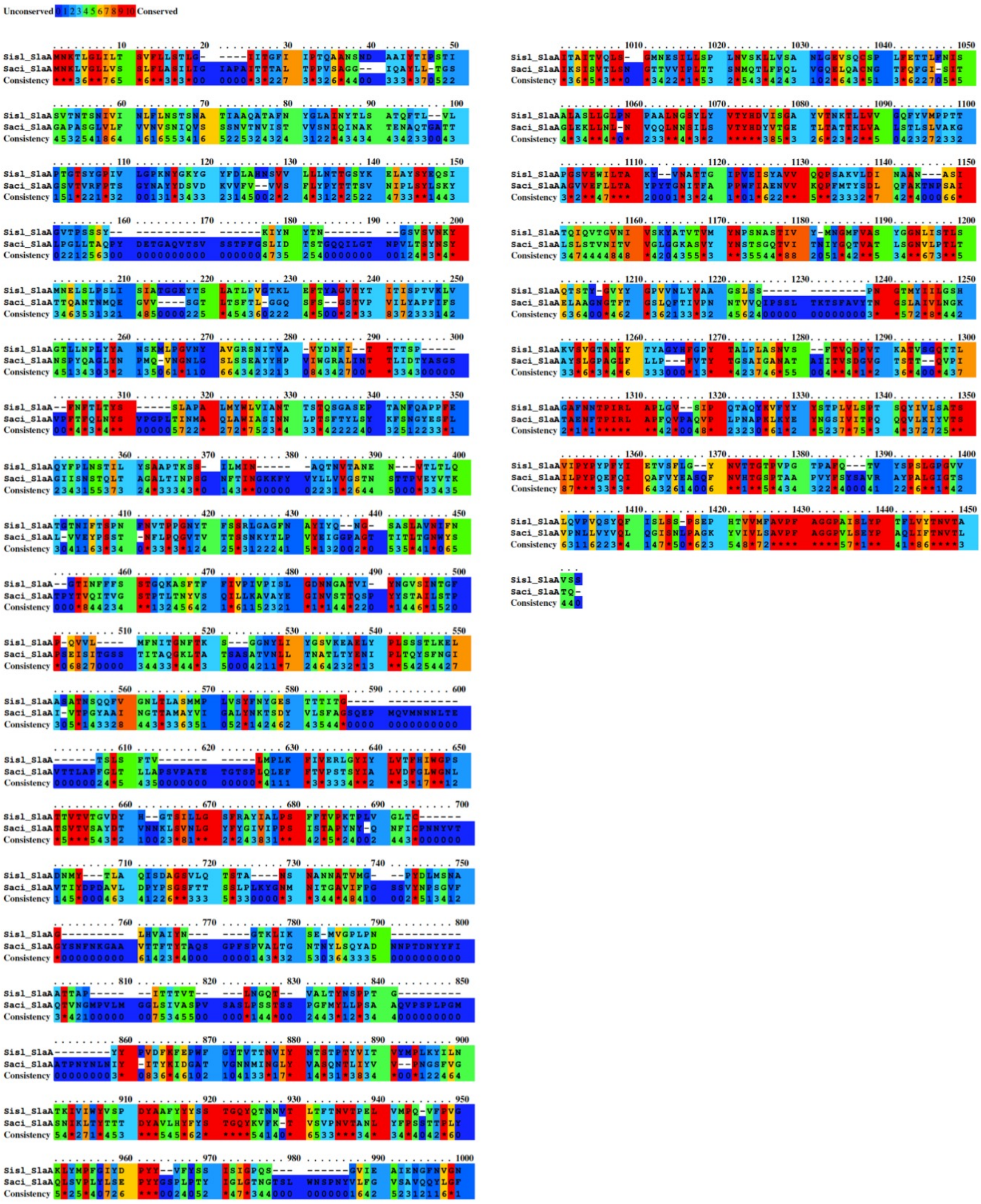

Fig S2
B

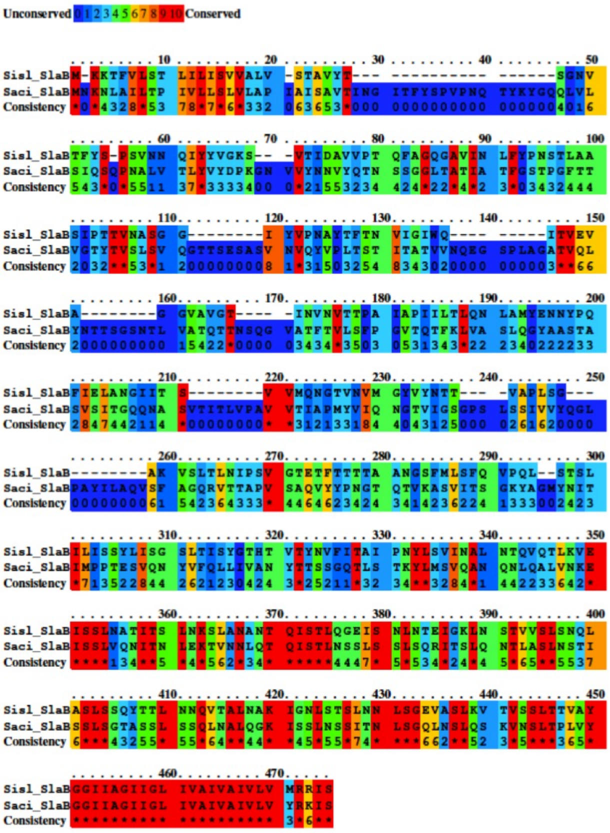
Unconserved
0 1 2 3 4 5 6 7 8 9 10
Conserved
Sisl_SlaB
Saci_SlaB
Consistency

Fig S2
C

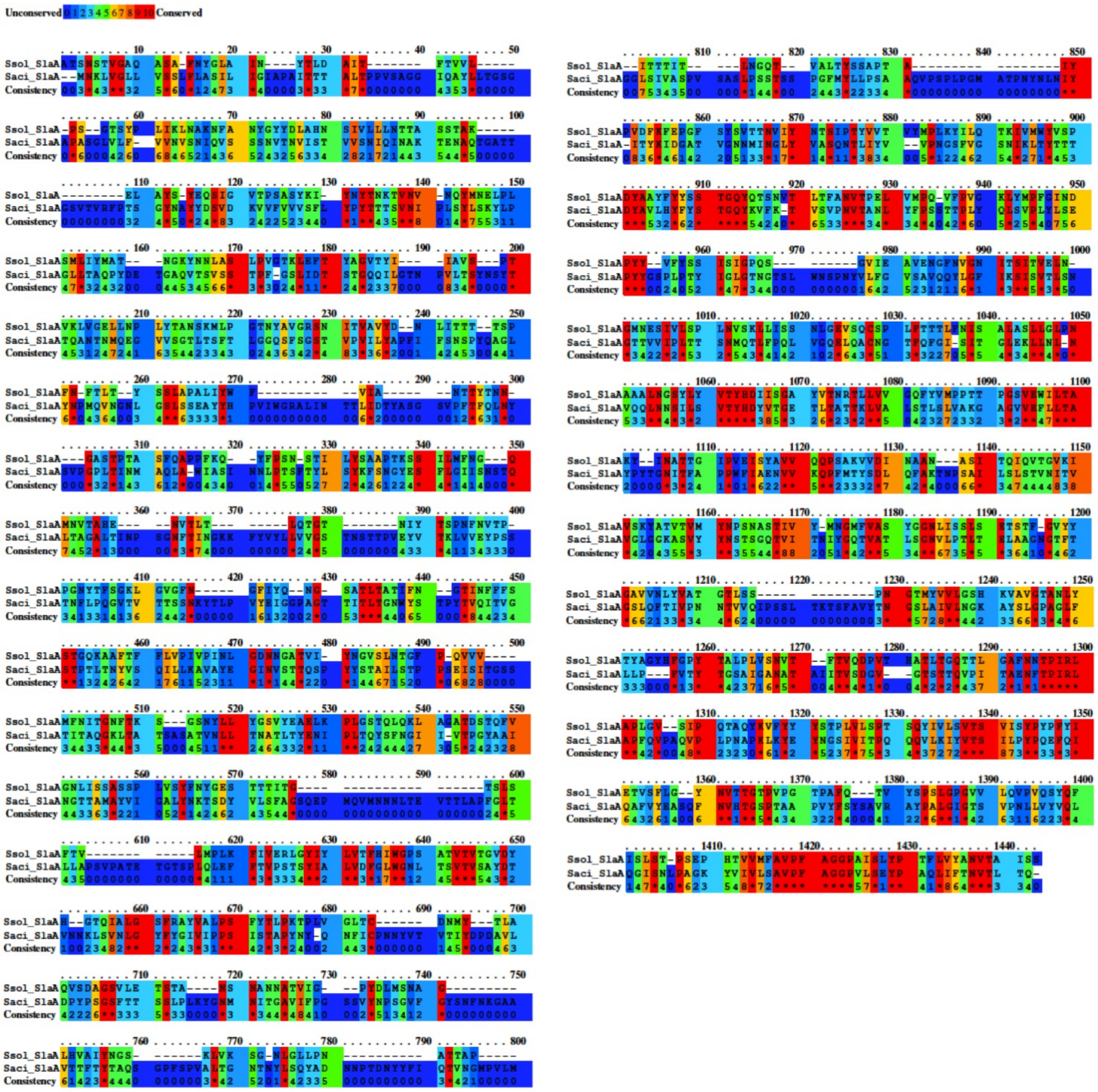

Fig S2
D

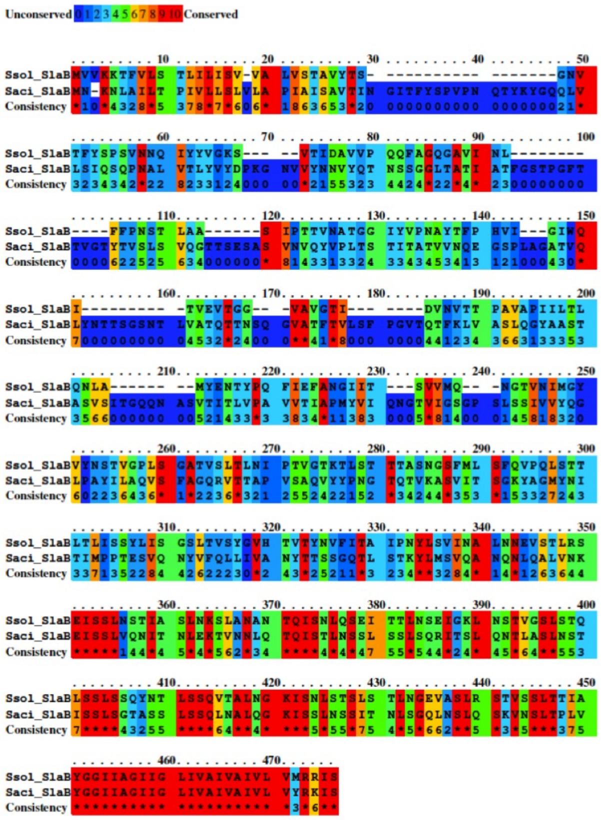

Figure S2 – Sequence alignment comparing SlaA and SlaB from *Sisl*, *Ssol* and *Ssaci*. The sequence identity: *Sisl*_SlaA / *Saci*_SlaA, 24% (A); *Sisl*_SlaB / *Saci*_SlaB, 25% (B); *Ssol*_SlaA / S*aci*_SlaA, 25% (C); *Sisl*_SlaB / S*aci*_SlaB, 26% (D).

Fig S3

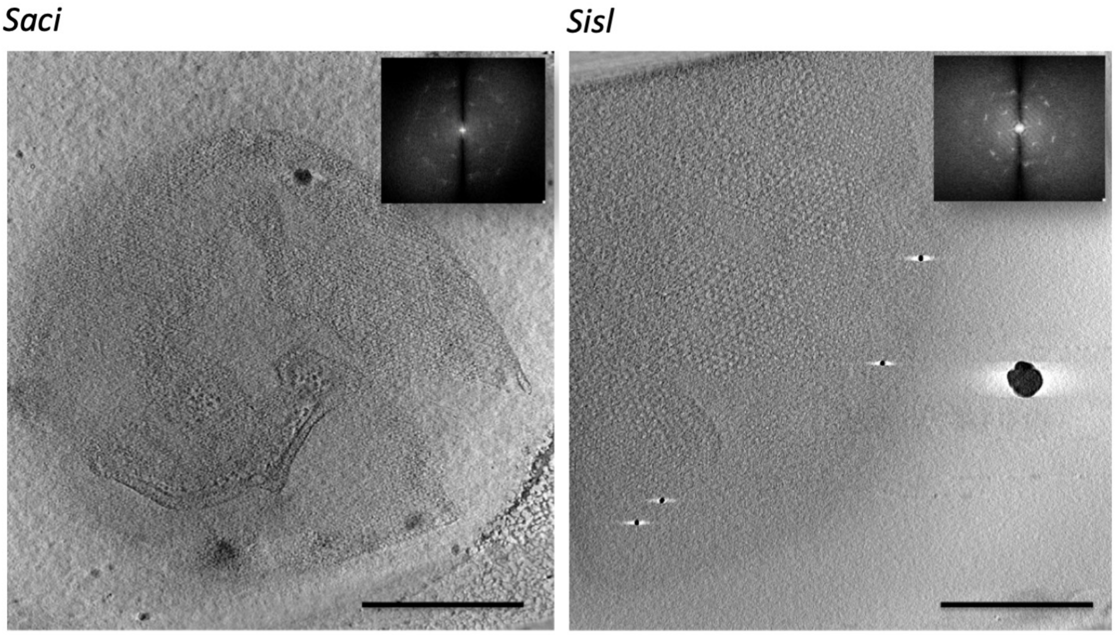

Figure S3 – Tomographic slices through S-layer planes in whole-cell tomograms. Left panel, tomographic slice through the S-layer of *Saci*; right panel, tomographic slice through the S-layer of *Sisl*. Insets, power spectra reveal 2D lattices with hexagonal symmetry.

Fig. S4

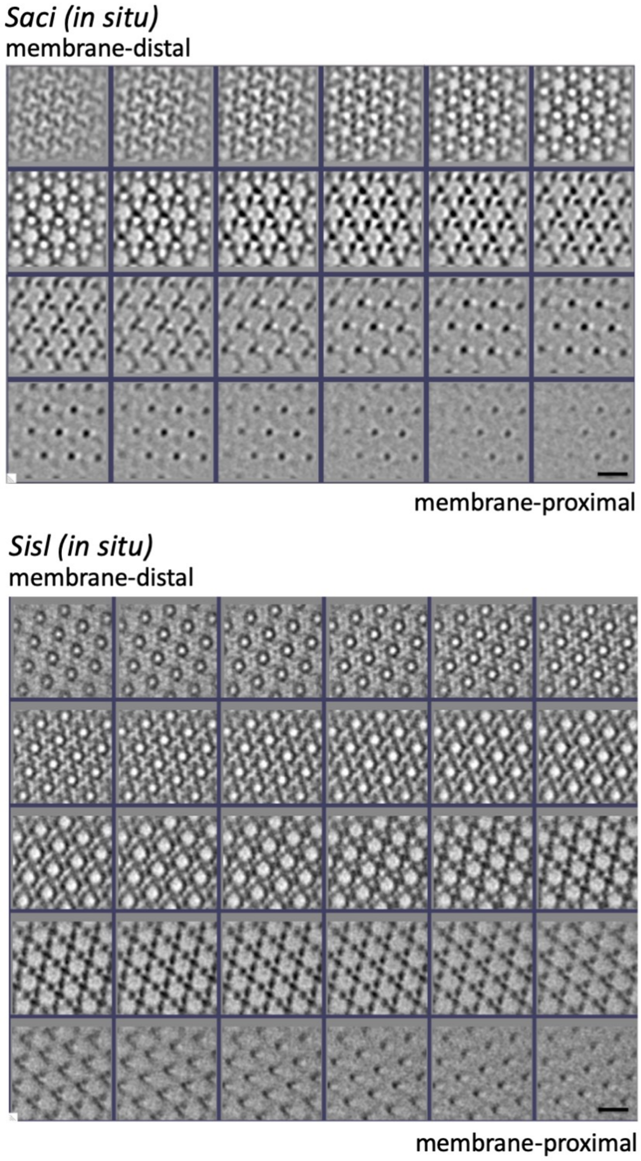

Figure S4 – Consecutive slices through sub-tomogram averages of S-layers. Top panel, in situ map calculated from whole-cell tomogram of *Saci*, bottom panel, map calculated from whole-cell tomogram of *Sisl.*

Fig. S5

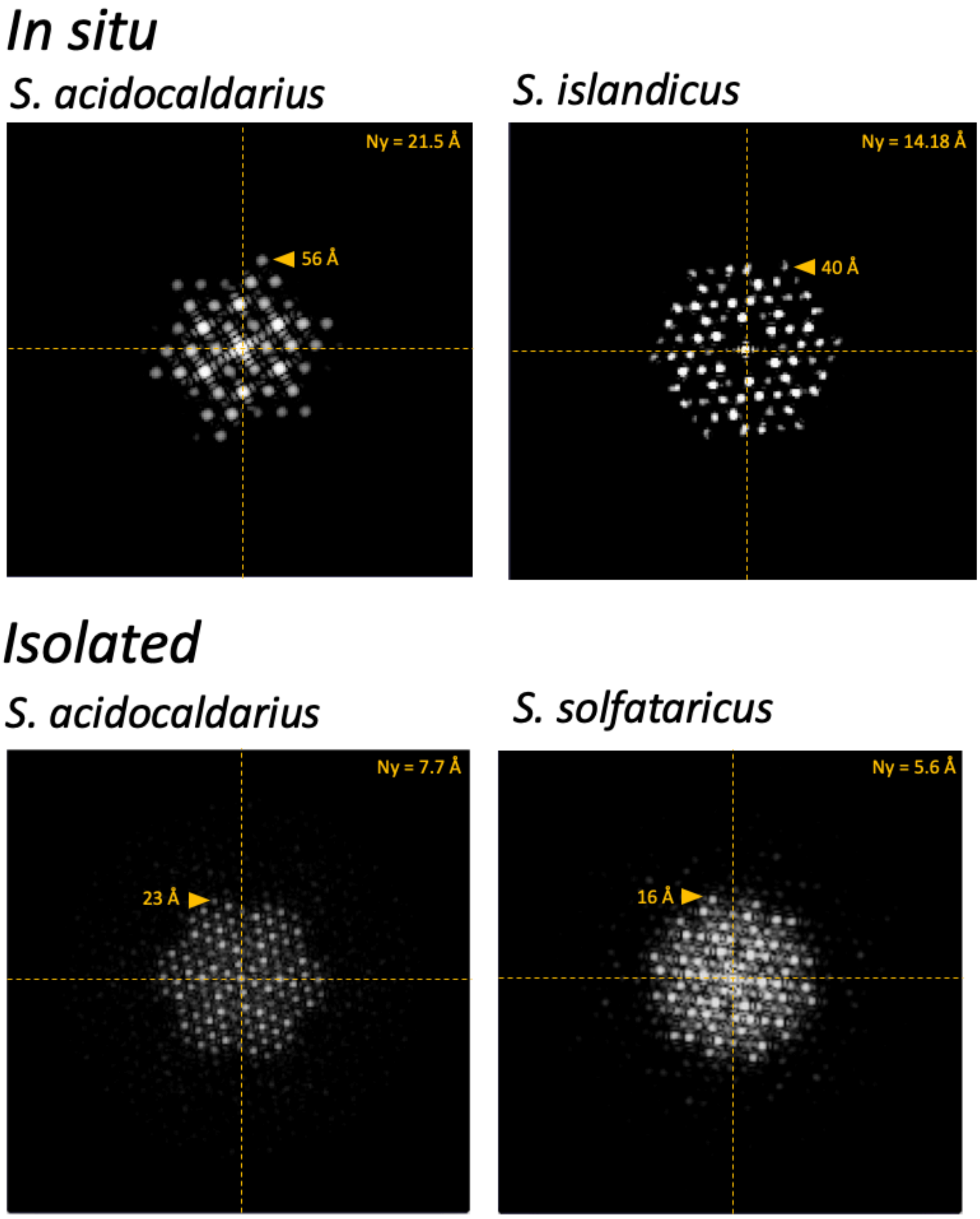

Figure S5 – Resolution of S-layer maps determined by power spectra. Top panel, power spectra of in situ maps calculated from whole-cell tomograms, bottom panel, power spectra of maps calculated from isolated S-layers. Ny, Nyquist.
